# Spread of *Dermacentor reticulatus* is associated with the loss of forest area

**DOI:** 10.1007/s10493-017-0160-8

**Published:** 2017-08-22

**Authors:** Ewa J. Mierzejewska, Agustín Estrada-Peña, Anna Bajer

**Affiliations:** 1 Department of Parasitology, Faculty of Biology, University of Warsaw, Warsaw, Poland; 2 Department of Animal Health, Faculty of Veterinary Medicine, University of Zaragoza, Zaragoza, Spain

**Keywords:** *Dermacentor reticulatus*, Range, Land cover, Temperature, Remote sensed data, Habitat modelling

## Abstract

Changes in tick distribution and abundance are among the main drivers of the (re)emergence of transmitted pathogens. We aimed to uncover the reasons of the reported spread of *Dermacentor reticulatus* in Poland using a variety of proxies of environmental features, ground-measured temperature and remotely-sensed data of temperature and vegetation. Ground-measured temperature was recorded in 2013–2014, in sites where *D. reticulatus* presence (n = 16) or absence (n = 16) was confirmed. We specifically aimed to test whether changes in phenology of vegetation and the length of the growing season were correlated with field-derived data regarding the presence/absence of *D. reticulatus*. We also used categorical descriptions of the habitat to capture the vegetation patterns that might delineate the distribution of the tick. We demonstrated that temperature, phenology of vegetation and length of growing season have no correlation with the occurrence of *D. reticulatus* in Poland. There is, however, a clear association between the trends of the loss of forests and the presence of the tick. This parameter was two times higher at sites colonized by *D. reticulatus* in comparison to the sites where the population of the tick is not yet established. A spatial analysis demonstrated that the preferred territories for *D. reticulatus* are those of a highly fragmented landscape within a large patch of homogeneous vegetation, in the vicinity of permanent water courses or reservoirs.

## Introduction

The emergence and re-emergence of tick-borne diseases (TBDs) pose a significant threat to both human and animal health (Jongejan and Uilenberg 2004; Solano-Gallego and Baneth 2011; Bajer et al. 2014). Appearance of a new TBD in non-endemic regions is often the first sign of the extending geographical range of a particular tick species (Nijhof et al. 2007; Schaarschmidt et al. 2013). Therefore, forecasting and early detection of the spread of ticks to new areas are needed for the prevention of TBDs (Otranto and Wall 2008).

Exophilous tick species spend most of their life questing for hosts in the environment and their survival is strictly dependent on abiotic conditions (Randolph 2009). A set of adequate conditions, delineated by microclimatic conditions such as slope and aspect, snow cover, vegetation, litter layer, humus, and underlying soils provides shelter for ticks during quiescence and diapausing and is appropriate for questing (Estrada-Peña 2008). All these variables are specific for each tick species. Completion of the life cycle and tick spread rely also on host availability, thus involving specific landscape and climatic conditions for vertebrates (Estrada-Peña and de la Fuente 2014). It is vital to capture the environmental features to which ticks are associated, given that the presence of probable suitable foci and spread of a particular tick species to non-endemic regions, could be modelled based on the features of preferred habitats (Kalluri et al. 2007; Eisen and Eisen 2008). For this purpose remotely-sensed (RS) data available on relatively long time scales have been used. However, they are commonly insufficient to describe the microclimatic conditions under which ticks thrive. The use of field-recorded environmental conditions (i.e. temperature, humidity) increases significantly the reliability of the analyses (Ogden et al. 2006a; Estrada-Peña 2008). These measures have been extensively used in the modelling of epidemiological risk related to ticks feeding on humans (Ogden et al. 2005; Hoch et al. 2010; Hancock et al. 2011).

Considerable attention has been devoted to *Dermacentor reticulatus* (Fabricius) in Europe in the last two decades (Dautel et al. 2006; Bullová et al. 2009; Dobec et al. 2009; Hornok and Farkas 2009; Cochez et al. 2012; Halos et al. 2013; Schaarschmidt et al. 2013; René-Martellet et al. 2015; Olivieri et al. 2016), given that it is the main vector of *Babesia canis* (Bourdoiseau 2006; Beelitz et al. 2012). This tick species may also constitute possible threat to public health as vector of tick-borne encephalitis virus (Wójcik-Fatla et al. 2011; Mierzejewska et al. 2015a) and bacteria of genus *Rickettsia* (Špitalská et al. 2012; Földvári et al. 2013). Poland is one of the areas of recent expansion of *D. reticulatus*. Until the end of the 1980’s, this species was recorded sporadically and was present in several restricted foci in eastern regions of the country (Lachmajer 1963; Szymański 1986; Siuda 1993). In the 1990’s, *D. reticulatus* became common in central Poland, including the area of the capital city, Warsaw (Karbowiak 2009; Zygner and Wedrychowicz 2006; Zygner et al. 2009), resulting in outbreaks of canine babesiosis in the region (Sobczyk et al. 2005; Welc-Falęciak et al. 2009). Until now this territory is considered as endemic for canine babesiosis (Adaszek et al. 2011; Bajer et al. 2014). The range of this tick species continued to expand during the last 20 years, crossing the Vistula River barrier (Karbowiak 2009, 2014; Zygner et al. 2009; Welc-Faleciak et al. 2009; Bajer et al. 2014). In endemic regions (central and eastern Poland) *D. reticulatus* is the dominant species of the tick fauna on pets and livestock, with a prevalence over 80% (Mierzejewska et al. 2015b). In 2010, the first focus of *D. reticulatus* was confirmed in western Poland (Nowak 2011) but the species had already been collected from animals in the Dolnośląskie (western Poland) and Pomeranian (northern Poland) voivodships (Fryderyk 1998; Karbowiak and Kiewra 2010). The occurrence of *D. reticulatus* in these regions was later confirmed in additional isolated locations (Kadulski and Izdebska 2009; Karbowiak and Kiewra 2010).

During the period of 2012–2014, we performed a large-scale active survey of *D. reticulatus* in the area between the Vistula River and the western border of Poland (Mierzejewska et al. 2016), where several new foci of *D. reticulatus* were found. We have confirmed the expansion of the tick range and noticed the presence of a large area between the Vistula and Oder Rivers which remains free of this tick species (the gap). In another study we have demonstrated that fallow lands (abandoned crop fields and meadows) are the preferred habitats for *D. reticulatus*, with tick densities up to eight times higher than in cultivated pastures and meadows (Mierzejewska et al. 2015c). The tick prefers open habitats close to periodically flooded zones in valleys of rivers and streams (Siuda 1993; Zygner et al. 2009; Široký et al. 2011).

It is however unknown what factors promote the spread of *D. reticulatus* tick and why some areas remain uncolonized, even if located within the invaded region. In this study we aimed to determine the features that shaped the recorded distribution of *D. reticulatus* in Poland. We compared actual (years 2013–2014) ground temperature profiles between sites where the tick is present (tick-positive site) or absent (tick-negative site). We also analysed long-term changes in environmental features (i.e. temperature, humidity, Normalized Difference Vegetation Index, forest habitat losses) between the tick-positive and the tick-negative sites, using RS data.

## Materials and methods

**Fig. 1:**
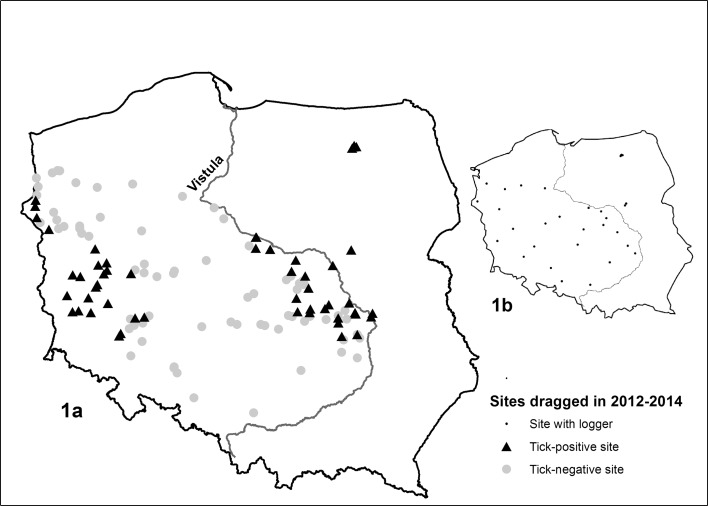
Location of the *Dermacentor reticulatus* positive and negative sites in the target territory in Poland (*1a*). Location of sites where loggers were placed to record ground temperature (*1b*)

All analyses were performed based on surveys carried out on 54 tick-positive and 74 tick-negative sites in endemic regions, the zones of expansion and the gap between endemic and colonized sites (Fig. 1a). We used the results of a survey of *D. reticulatus* in Poland, performed in 2012–2014. The survey (selection of tick collection sites and their locations) has been described in detail in Mierzejewska et al. (2016). Nine tick-positive sites were situated in the endemic territory: four in eastern (Warmińsko-Mazurskie) and five in central (Mazovia) Poland. Twenty-one sites were located on the western side of the Vistula River, in the zone of expansion (ZE) of the tick. There were 24 sites in western Poland, including two in the Lubuskie focus described by Nowak (2011). The sites negative for *D. reticulatus* were located in both the ZE and the gap.

### Weather conditions among the tick-positive and tick-negative sites

#### Temperature at ground level

A total of 32 loggers were used for the measurement of ground temperature (HOBO U23-001 Pro v2 Temperature/RH Data Logger, Onset Computer Corporation, USA). Twenty-five loggers were regularly distributed in the ZE and the gap (Fig. 1b). Seven additional loggers were placed in the endemic region on the eastern side of the Vistula River. In 16 of these 32 sites, the presence of *D. reticulatus* was confirmed. Loggers were buried 5 cm below ground level in perforated plastic boxes, used for protection. The temperature was recorded four times a day (at 04:00, 10:00, 16:00 and 22:00) from the middle of November 2012 to the beginning of July 2014. We first compared the monthly average temperature among the tick-positive and negative sites. Similar comparisons were also done with the total amount of temperature accumulated in monthly calendar intervals. For both analyses we used nested ANOVA (JMP V9.0 software) to test for statistically significant differences between the tick-positive and negative sites for any specific month, given that climatic conditions may have varied effects on ticks at specific life cycle stages (Zahler and Gothe 1995a, b; Randolph 2009).

#### Remotely sensed data

We used RS data to search for differences in the long-term trends of environmental features between the tick-negative and the tick-positive sites. A set of Fourier-transformed RS covariates (Estrada-Peña et al. 2014) was used to test differences in land surface temperature during the day (LSTD) and night (LSTN) as well as in the vegetation (Normalized Difference Vegetation Index, NDVI) values between the tick-positive and tick-negative sites. NDVI is a measure of the photosynthetic activity of the vegetal canopy (Wan et al. 2004) that is related to the transpiration of the plant and to its water contents. Monthly data on LSTD, LSTN, and NDVI at a spatial resolution of 0.05° were obtained from the MODIS website (https://lpdaac.usgs.gov/products/modis_products_table, accessed in December 2013). The data covered the period of 2001–2012. A complete explanation of the procedures for harmonic regression and extraction of the coefficients, as well as their improved performance over monthly raw series of RS data were provided earlier (Estrada-Peña et al. 2014). The coefficients were calculated separately for LSTD, LSTN, and NDVI, and therefore three different multifactorial ANOVA analyses were carried out to check for consistent differences in long-term RS values recorded at the tick-positive or negative sites. Given that this is a spatial analysis, point coordinates of the surveyed sites are not adequate. We therefore drew hexagons with a radius of 5 km around each collection site, and remotely-sensed features overlying each hexagon were averaged for every collection site. This set of hexagons was used for the further spatial analysis described below.

### Vegetation phenology

#### Vegetation, land cover and its trend

We explored if medium-term changes in vegetation phenology exist between the tick-positive and negative sites on the basis that significant differences of the trends of vegetation would likely reflect different environmental conditions for *D. reticulatus*. We used the Global Inventory Modelling and Mapping Studies (GIMMS) NDVI data set obtained from http://ecocast.arc.nasa.gov/data/pub/gimms/. The attributes of the data set were as follows: 15-day temporal frequency, 250 m spatial resolution and a temporal span from January 1982 to December 2006. This is the latest available update of the dataset; information between 2007 and 2014 is not yet available. We examined the inflection points of the complete GIMMS time series to examine where inflection points exist using the package “cpm” (Ross 2013; http://cran.r-project.org/src/contrib/Archive/cpm/, accessed in January 2014) for the R programming environment (R Core Team 2014). We assessed significant differences in the inflection points of the NDVI series between the tick-positive and negative sites, using ANOVA as in the previous analysis.

#### Changes in the length of the growing season

The GIMMS NDVI data set was used to assess changes in the length of the growing season. It has been demonstrated that the success of colonisation by *D. reticulatus* may be highly dependent on the length of the oviposition period, which happens in spring and summer (Zahler and Gothe 1995a, b). Thus, the fitness in the oviposition period could account for the success of tick colonisation (Dautel et al. 2006) because the longer the growing season, the higher the temperatures (Jaenson and Lindgren 2011), and therefore the better conditions for the oviposition by the tick. We checked for significant differences in the duration of the growing season between the tick-positive and negative sites, using the package “changepoint” (Killick and Eckley 2014) for R. The hypothesis is that the length of the growing season for the complete series 1982–2006 would show significantly different values between the tick-positive and negative sites. Because of the uncertainties in this analysis, we completed it in two different ways: one which included the number of 15-day periods between spring and autumn, and the other including only the number of 15-day periods of summer. Next, we analysed the amount of the NDVI accumulated between the beginning and the end of the considered seasons, looking for further significant differences between the tick-positive and negative sites, using, as before an multifactorial ANOVA.

### Changes of landscape as associated with the presence/absence of *D. reticulatus*

Ticks are sensitive to changes in the landscape (Estrada-Peña 2003; Ostfeld et al. 2005). This is sometimes complex to evaluate because ticks are not associated to pre-tailored categories of vegetation, that are based on purely botanical terms (Gilot et al. 1979; Hubálek et al. 2003). Therefore, we used a combined analysis involving both the changes in forest areas and in the types of vegetation, adhering to the methods explained below.

#### Vegetation Continuous Fields

We used the Vegetation Continuous Fields (VCF) dataset (DiMiceli et al. 2011), for the period 2000–2010 at a spatial resolution of 250 m (available at http://glcf.umd.edu/data/vcf/, accessed in December 2013) to test for changes of “forest” or “grassland” in the target territory. Given that *D. reticulatus* prefers open sites, we tested for differences in VCF trends between the tick-positive and negative sites, which should ascertain trends in the loss of forest habitats. Trends for the VCF series and statistically significant differences were computed as earlier.

#### Spatial analysis of landscape categories: High-resolution habitat categories

We assessed if *D. reticulatus* is associated with a range of categories of vegetation, as defined by harmonised high-resolution datasets, and if the differences between the tick-positive and negative sites are statistically significant. We used the GlobCover dataset, produced by the European Space Agency (Bicheron et al. 2006, available at http://www.gofcgold.wur.nl/sites/globcover.php, accessed in December 2013). It is a set of 40 categories of vegetation at a resolution of 300 m, developed from the MERIS instrument on board the ENVISAT series of satellites. Attempts to use the CORINE dataset produced unreliable results, because such a dataset is currently available only at level 3 for Poland. The low number of landscape categories associated with CORINE 3 resulted in an insufficient description of the preferred habitat for *D. reticulatus*.

We associated the type of dominant vegetation, the fragmentation (number of different categories of vegetation) and the distance to either water courses or reservoirs to the tick-positive or negative sites. This is in part a qualitative assessment (as opposed to quantitative) of the preferred habitat of the tick, and therefore statistical tests accounting for quantitative variables cannot be applied. We therefore used a recursive partitioning method to uncover the set of variables that better split the surveyed points into the tick-positive or the tick-negative sites. The method recursively partitions data according to relationships between the explanatory variables and the response variable, here the tick-positive/negative site, creating a tree of partitions. It finds a set of cuts or groupings of X values that best predict an Y value. It does this by exhaustively searching all possible cuts or groupings. These partitions of the data are done recursively, forming a tree of decision rules until the desired fit is reached. Node splitting is based on the LogWorth statistic (Gaudard et al. 2006). After the algorithm recursively splits all the possible combinations of the explanatory variables, a final decision tree is available, which displays the partitions and the best combination of variables that separate the tick-positive or negative sites for *D. reticulatus*. We used a nested ANOVA to check for statistical differences between the different type of vegetation (using the mentioned different datasets) in the surveyed territory.

## Results

### Effect of weather features on the successful colonisation by *D. reticulatus*

#### Temperature at ground level

**Fig. 2:**
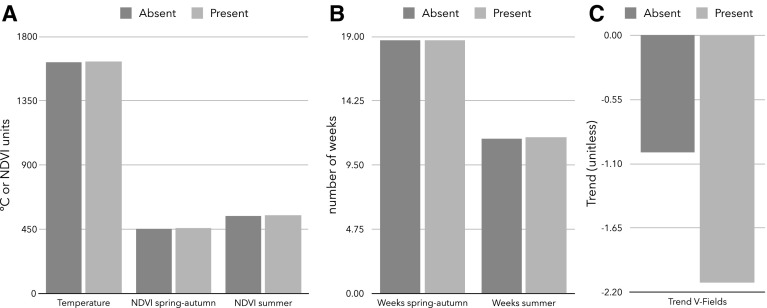
A comparative overview of the main quantitative traits measured to ascertain the distribution of *Dermacentor reticulatus*. **a** Values of the monthly accumulated temperatures (°C) over the year for the *D. reticulatus* positive and negative sites and average of monthly accumulated NDVI values in the period 1982–2006 for *D. reticulatus* positive and negative sites in the target territory. Regarding NDVI, the chart shows the accumulated values of NDVI in the periods which were detected as belonging to “summer” or to “spring to autumn” by an algorithm that explores the inflection points of the time series of data. Original data were obtained from the GIMMS-NDVI dataset. **b** Number of 15-day intervals which were detected as belonging to “summer” or to “spring to autumn”. Data regarding NDVI were obtained from the Global Inventory Modelling and Mapping Studies (GIMMS)-NDVI dataset. **c** Differences in the trend of vegetation fields values between positive and negative sites for *D. reticulatus*. The index indicates the “amount of forest”, therefore, the lower the value, the highest the trend in the time for vegetation stands belonging to the category “forest” that were transformed into “open vegetation” in the period of time

We found no significant differences in the annual temperature profiles at ground level between the tick-positive and negative sites. In fact, the monthly average temperature for both site categories overlapped (Fig. 2a).

#### Remotely sensed data

The analyses of Fourier-transformed RS covariates for LSTD, LSTN and NDVI in a 12-year time span did not show any significant differences between the tick-positive and negative sites (*p* = 0.37 for all the variables).

### Vegetation phenology

#### Land cover and its trend using the GIMMS-NDVI data set

The NDVI calculated for the 25-year data set showed a general positive trend. However, differences in the NDVI trend between the tick-positive and negative sites were not significant.

#### Changes in the length of the growing season

No significant differences were evident in the comparison of the duration of spring–autumn, the duration of summer, the accumulated NDVI in spring–autumn and in the summer (Fig. 2b).

### Changes of landscape as associated to the presence/absence of *D. reticulatus*

#### Vegetation Continuous Fields

The analysis of the trend of VCF in the period 2000–2010 showed that the loss of forest habitats for the tick-positive sites was twice as high as compared to the tick-negative-sites (Fig. 2c) (*F*_1,11_ = 13.45; *p* = 0.01).

#### A spatial analysis of landscape qualitative categories

**Fig. 3:**
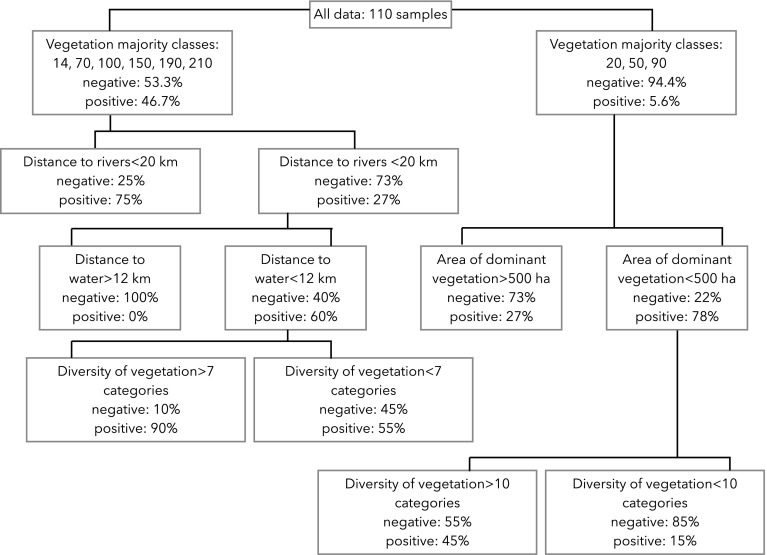
Results from a recursive algorithm that partitions categorical descriptions of the habitat, aimed to discriminate the sites positive or negative for *Dermacentor reticulatus*. The algorithm looks for the combination of variables that best discriminate two categories of sites and produces the most adequate separation between the tick-positive and negative sites. The algorithm is recursive and separate sites not well discriminated using further combinations of variables

We assessed the reliability of the combined analysis of the diversity of vegetation, the dominant category of vegetation, and the distance to rivers or to water courses, to assess the presence or absence of *D. reticulatus* (Fig. 3). No single or set of variable(s) correctly classified a tick-positive or tick-negative site. The best model, measured with the Akaike Information Criterium, was 318.8, with an r-square of only 0.33 with a total of seven splits of the categorical variables. This model ascribed the majority of the records of *D. reticulatus* to cells in the grid displaying a large single patch of vegetation surrounded by a highly fragmented landscape, in the vicinity of a permanent water course or reservoir.

## Discussion

This paper explored the reliability of several vegetation and weather-derived data (including landscape classifications) to separate the sites where *D. reticulatus* is present or absent in a wide territory of Poland and to conclude on the possibility of further expansion. We found very few statistically significant associations, supporting the idea that the tick is colonizing pre-existing suitable habitats. Perhaps surprisingly, variables used before to describe the habitat or niche of ticks (Mejlon 1997; Vail and Smith 1998; Alekseev and Dubinina 2000; Schulze et al. 2001) did not help to define *D. reticulatus* distribution in Poland. Ground-measured and RS-derived temperature do not describe the pattern of occurrence of *D. reticulatus* in Poland.

Similar studies for *D. reticulatus* are scarce and focused to capture the periods of activity and abundance of the tick (Hubálek et al. 2003; Bartosik et al. 2011; Buczek et al. 2014). The comparison of features determining the limiting factors for its occurrence pattern in a large territory has never been done before. Hubálek et al. (2003) investigated the impact of microclimate in South Moravia (Czech Republic) on the adults of *D. reticulatus*. The soil temperature was found to be the variable with the highest importance in the predictive models of the relative abundance and host seeking activity of *D. reticulatus*. The temperature threshold for questing behaviour of adults was around 0.7/–0.1 °C, explaining its activity period in autumn and winter. In our study, the two series of ground-recorded and RS measurements of temperature gave coherent results, without differences between the tick-positive and negative sites. This is an important finding, given that satellite data are available for analysis since the beginning of the twenty-first century; therefore it covers the time span during the expansion of *D. reticulatus* in Poland. The lack of significant differences between the tick-positive and negative sites leads to a more general conclusion regarding the likely non-significant impact of temperature trends on this tick species. This underlines the need for further investigation to determine other possible reasons for the observed expansion. The main conclusion from this part of the analysis is that, irrespective of the changes in weather over the last few years, there are no significant differences between the sites colonised by ticks and those that are still free of *D. reticulatus*.

The analysis of a similar series of values of NDVI was unable to detect changes in the photosynthetic activity of the plants in the sites where the tick is present or absent. NDVI is a convenient surrogate to evaluate changes and trends of humidity in the vegetation canopy. NDVI is only temporally responsive to rainfall, and is more suitable for a long term measurement of water contents, which in turn is known to affect tick activity and mortality (Randolph 2000; Sonenshine and Roe 2014). NDVI has been repeatedly used to monitor the habitat of ticks (Estrada-Peña 1999; Bisanzio et al. 2008; Ogden et al. 2006b). Hubálek et al. (2003) and Buczek et al. (2014) have demonstrated the lack of impact of rainfall and wind speed on the host-seeking activity of *D. reticulatus*. Rainfall is unlikely to be an important factor limiting the distribution of *D. reticulatus* in Poland, given that annual mean precipitation is 622.8 mm (data from the Institute of Meteorology and Water Management) with the lowest values in the centre of the country (about 550 mm) and the highest in mountain regions (about 1000 mm). The optimal range of annual mean precipitation for *D. reticulatus* (400–1000 mm) was estimated by Gilot et al. (1973). In summary, the two most important traits of the weather, namely temperature and air water contents, did not differ between the colonised sites and those where the tick has not yet been collected in Poland.

We thus turned to examine if the growing season changed between the two categories of sites. The rationale is that changes in the duration of the growing season could be a more adequate marker of habitat suitability for *D. reticulatus* because a seasonal component is included. This is of importance because averaged values of weather variables tend to obscure the importance of a given trait at critical moments of the life cycle of the tick. This approach, however, also failed to provide significant differences between the tick-positive and negative sites in the surveyed territory. While obvious changes in the duration of summer or spring–autumn were noted, as already reported (Fensholt et al. 2009; Fensholt and Proud 2012) they do not correspond with the pattern of occurrence of the ticks. We think that high resolution data, such as a long term series of Landsat images, could probably increase the sensitivity in detecting changes in the trend of NDVI for tick presence/absence sites. A major limitation in the use of this approach is to cover a large territory with images that have a resolution of only 15 m, and for which a large number of images are necessary to cover the period of interest. Landsat images, however, have been used in studies defining the habitat of *Amblyomma variegatum* (Hugh-Jones 1991) and in delineating areas according to the force of transmission of tick-borne diseases (Daniel et al. 1998).

The results of the trends of VCF analysis indicate that this series may be a promising indicator of the habitat for *D. reticulatus*. This set of data is intended to deliver information about the trends of changes in forested versus open habitats. We demonstrated that the increase in the trend of the loss of forest habitats was twice as high for the tick-positive sites as for the negative ones in the complete target territory. The time span of data availability (2000–2010) was relatively short, thus greater differences between the tick-positive and the tick-negative sites are expected. Forests are most often transformed to arable land, or lost because of urbanisation or to lesser extent as a result of forest management (Firbank et al. 2008). These factors promote the creation of open habitats, preferred by *D. reticulatus*. The development of a mosaic landscape, in which forest has a trend to slowly disappear, would favour the occurrence of the tick both because it provides suitable set of conditions for *D. reticulatus* and the landscape favours the presence of hosts.

We aimed to integrate these findings into a classification of the territory that includes the amount of forest, the fragmentation of the habitat, and the distance to rivers and water courses. The latter is based on the reported findings that large vertebrates tend to use water courses for movements (Dickinson et al. 2000) and provides a proxy for “host availability” and connectivity among tick infested patches. The results showed the reliability of this approach to characterise the sites colonised by *D. reticulatus*. The method, however, did not give a complete and perfect separation of sites, probably due the low number of descriptive levels of the available dataset (3 levels as standard for Europe). CORINE Land Cover at 5 levels would probably increase the predictive power of this analysis, but it is currently unavailable for Poland. Results of the landscape analysis indicate that areas preferred by *D. reticulatus* are large patches of homogeneous vegetation surrounded by a highly fragmented landscape, in the vicinity of a permanent water course or reservoir. This is a heterogeneous mosaic that may be highly interconnected, allowing the movements of infested hosts and therefore improving the ability of the tick to colonise new sites (Li et al. 2012; Kittle 2014). A landscape in which the forest is progressively cleared and fragmented seems to be the preferred habitat for *D. reticulatus*, also in terms of the mobility of hosts and their essential role in the transportation of ticks (Jaenson et al. 2012; Carpi et al. 2008; Pugliese and Rosa 2008; Hoch et al. 2010). Populations of wolves and moose, which have a great mobility (Mech and Boitani 2003; Hoffman et al. 2006), have recently increased in Poland and moose is considered as one of the main hosts of *D. reticulatus* (Bogdaszewska 2004). Water courses support landscape connectivity serving as potential corridors for wild animal migration. Banks of rivers and canals also provide open land with shrubs and high grasses described previously as a preferred habitat of *D. reticulatus* (Siuda 1993; Zygner et al. 2009; Široký et al. 2011).

## Conclusions

Results of this study indicate the temperature (recorded at micro and macro scale) and length of the growing season create no barrier for the spread and distribution of *D. reticulatus* in the territory of Poland. However the spread of *D. reticulatus* tick is associated with the loss of forest.
